# Supplementary material for: Are carbon emissions trading and green financial instruments synergistic? -Comprehensive quantitative research based on content analysis
Source: PLoS One. 2024 Mar 7;19(3):e0298601. doi: 10.1371/journal.pone.0298601 (PMC10919601; doi:10.1371/journal.pone.0298601)
Supplement: S1 Data — (ZIP) [file pone.0298601.s001.zip › Data/地级市Z_F工作报告.zip!/╣π╢1⁄2/╤⌠╜¡╩╨/└┤╫╘╣1⁄2╓┌║┼í╛┬φ┐╦╩2╛▌═°í┐_2016.docx]

各位代表：

我代表市人民政府向大会作政府工作报告。请各位代表连同《阳江市国民经济和社会发展第十三个五年规划纲要(草案)》一起审议，并请政协各位委员和其他列席人员提出意见。

一、“十二五”时期及2015年工作回顾

“十二五”时期，是阳江经济社会发展极不平凡的五年。在市委的正确领导下，在市人大及其常委会和市政协的监督支持下，市政府全面贯彻落实中央、省的决策部署，紧紧围绕主题主线和“在加快发展中转变方式，实现幸福追赶”的核心任务，深入实施“双化”驱动、蓝色崛起和对接珠三角战略，突出抓好“三大抓手”，经济社会发展取得显著成就，较好地完成了“十二五”规划确定的目标任务。

过去五年，科学发展成效显著、经济实力明显增强。全市地区生产总值年均增长12.4 %，2011至2014年增速连续4年位居全省前三名。人均生产总值年均增长11.4%，2013年在粤东西北地区率先超过全国同期平均水平。一般公共预算收入比2010年翻一番多，年均增长21.4%。固定资产投资年均增长20.5%。外贸进出口年均增长10.1%。社会消费品零售总额年均增长13.2%。

过去五年，产业结构不断优化、转型升级步伐加快。三次产业比例由2010年的22∶41.8∶36.2调整为16.4∶47.3∶36.3。电力能源、金属压延等成为主导产业，五金刀剪产业年产值突破500亿元，规模以上工业总产值、增加值年均分别增长22.7%和23.7%。旅游、商业综合体等一批项目建成，港口物流、专业市场等加快培育。建成了一批现代农业园区、畜禽和水产品生产基地，海产品总量和人均占有量居全省前列，农业总产值年均增长3.7%。

过去五年，城市空间有效拓展、城乡面貌焕然一新。阳江滨海新区全面加快建设，完成阳东撤县设区，中心城区面积从44平方公里扩大至64.05平方公里，全市城镇化率比2010年提高3.2个百分点，成功创建国家园林城市。县城、中心镇辐射带动能力增强，一批美丽乡村建成。“一港两桥三路”等重要交通基础设施建设扎实推进，新增公路通车里程1240公里，建成万吨级以上码头4个。完成110千伏及以上输变电工程22项、水利防灾减灾工程133宗。“十二五”节能减排任务完成，森林覆盖率达58.2%，城市环境空气质量位居全省前列，漠阳江水环境质量总体达国家Ⅱ类标准，入海河口和近岸海域水质均符合功能区水质标准，连续4年获省环保责任考核优良等次。

过去五年，改革开放积极有为、发展活力有效释放。市、县新一轮政府机构改革完成，市级取消、下放和转移行政审批事项338项。建成省网上办事大厅阳江分厅和县(市、区)分厅。在全省率先成立市检测检验中心、公共资源交易中心和应急指挥中心。在全省非试点地区率先实施商事制度改革。组建了四大国有企业集团。医疗卫生体制改革深入推进，医疗资源整合成效明显。居民阶梯电价和市区居民生活用水、生活用管道天然气阶梯价格改革完成。五年新增社会组织997个。农村土地承包经营权确权登记颁证工作全面展开，集体经济收入10万元以上行政村的政经分离改革完成。

过去五年，社会事业全面进步、人民得到更多实惠。民生投入不断加大，办好每年十件民生实事。人民生活水平逐年提高，城乡居民人均可支配收入达17940元。投入23.3亿元新建和改扩建中小学校，基本完成创建教育强市任务。在全省率先组建市公共卫生医院，全市医疗机构床位数增长42.1%。建成一批文体惠民工程，提前两年实现乡镇农民体育健身工程全覆盖，全部公共文化场馆(站)免费向社会开放。城镇登记失业率控制在3%以内。城乡居民医保和养老保险基本实现全覆盖。建成公租房8263套，改造建设农村低收入住房困难户住房25393户。完成两轮扶贫开发“双到”工作任务。

刚刚过去的2015年，市政府积极应对经济下行压力，全力稳增长、调结构、促改革、惠民生、防风险，经济社会平稳发展，总体完成了市六届人大五次会议确定的目标任务。

经济保持平稳增长。主动适应经济发展新常态，采取系列措施推动经济增长。预计全市实现地区生产总值1256亿元，增长8.5%;人均生产总值50000元，增长7.6%;一般公共预算收入67.92亿元，增长6.2%。扩大有效投资，固定资产投资689亿元，增长4%。培育消费增长点，社会消费品零售总额582亿元，增长9.5%。加大外贸扶持力度，进出口总额178.8亿元，增长8.3%。促进金融健康发展，金融机构本外币存款余额1014.74亿元、贷款余额757.74亿元，分别增长11.4%和7.2%。

结构调整扎实推进。加快工业转型升级，阳江核电站2、3号机组等项目建成投产，阳江抽水蓄能电站、新兴铸管等项目动工建设，投入2.16亿元财政资金加快企业新一轮技术改造，工业六大支柱产业和先进装备制造业平稳较快发展，全市规模以上工业总产值2017亿元、增加值491亿元，分别增长12.3%和13.5%。抓好“1+10”专业招商和先进装备制造业招商，新引进维达生活用纸等项目130个，总投资429.8亿元。促进产业园区扩能增效，4个省级园区共投入10.7亿元完善基础配套设施，实现工业总产值1001.7亿元，增长21.9%。加快发展现代服务业，海陵岛大角湾海上丝路旅游区成功创建为全省首个滨海类国家5A级景区，休闲旅游业加快发展，全市接待游客1658.6万人次，旅游总收入181.3亿元，分别增长10.7%和18%。市电商创业园建成运营，首家公用型保税仓库挂牌。积极发展现代农业，创建市级现代农业园区11个，新增省级示范性家庭农场16户、省级农民专业合作社示范社23家，农业总产值344亿元，增长3.5%。新增钢质渔船71艘，渔业总产值147亿元，增长3.1%。提高科技创新能力，建成阳江五金刀剪生产研发基地和五金刀剪产业技术研究院，设立首家省部产学研合作院士工作站，新增国家级高新技术企业3家、省级工程技术研究中心5家。

城乡建设品质提升。阳江滨海新区核心区13个市政道路项目和13个商住项目加快建设，漠阳湖公园(一期)等项目动工，市妇幼保健院新院主体工程完成。阳东城区道路、环卫等市政设施进一步完善，市区一体化进程加快。深入创建国家卫生城市和国家环保模范城市，三江河涌环境综合整治、市区雨污分流管网改造工程基本完工，市区城北污水处理厂动工建设，阳江森林公园(一期)等工程加快推进，违章建筑整治和市容市貌“百日整治”取得实效，“三旧”改造扎实开展，城市环境不断改善。推进宜居城乡建设，阳春市创建为省园林城市，合水镇、合山镇分别被评为省宜居示范镇和名镇，19条村被评为广东省名村，120条美丽乡村建设任务基本完成。不断完善城乡基础设施，深茂铁路阳江段、云阳高速公路阳春段、汕湛高速公路阳春段加快推进，海陵岛大桥动工建设，省道371线阳春三甲至双滘段改造等工程建成通车。110千伏圩河输变电等项目建成。漠阳江双捷引水工程拦河闸重建动工建设，加固达标海堤50公里。加强生态环境保护，漠阳江流域整治扎实推进，11个中心镇污水处理厂主体工程基本完工，阳春市生活垃圾填埋场投入使用，那格电镀城搬迁后改造整治(一期)完成，淘汰黄标车5988辆，造林6.18万亩，单位生产总值能耗下降3.57%。

改革开放不断深化。加快转变政府职能，公布38个市直部门权责清单，完成市级非行政许可审批事项清理，市政府28个部门分别整合设置行政审批科。镇(街道)行政体制改革扎实推进。抓好投资管理体制改革，企业投资项目准入负面清单全面实施。实行“三证合一、一照一码”登记管理模式，新登记市场主体18920户，增长10.1%。市直及各县(市、区)公务用车、县以下机关公务员职务与职级并行制度改革基本完成。推进事业单位改革，市卫生系统4个事业单位完成干部人事制度改革。农村产权交易平台建设基本完成。加强区域合作，珠海阳江对口共建在产业园区建设、联合招商、民生合作等方面取得显著成效。

民生福祉持续改善。推进基本公共服务均等化，全市公共财政民生支出88.03亿元，增长23.1%，占财政预算支出的59.6%。十件民生实事全面完成。加强就业工作，新增城镇就业42468人，转移农村劳动力50275人。不断完善社会保障体系，城镇职工五大险种参保人数141.6万人次，城乡居民医保和养老保险待遇、低保救助、五保供养标准等达标提高。基本建成公租房588套，改造农村危房5144户、华侨农场危房236户、棚户区住房250户，建成因灾“全倒户”新居270户。抓好教育创强工作，阳东、阳春和阳西创建为广东省教育强县(市、区)。加强卫生计生工作，成立市人民医院医院集团，落实镇卫生院“五个一”设备配置，人口计生工作有效落实。推进文体惠民，建成10个基层公共文化体育综合服务示范点，举办了南国书香节暨第四届阳江书展、广东国际风筝邀请赛、群众文化节暨海上丝绸之路文化展演等大型文体活动。“南海I号”全面发掘保护和参与申遗工作扎实推进。改善公共交通，新增118辆新能源公交车。第二轮扶贫开发“双到”工作成效明显，105条重点帮扶村年集体经济收入平均达9.14万元，有劳动能力的贫困户年人均纯收入达10321元。做好“5+5”社会矛盾化解，深入开展公安机关“3+2”专项打击整治行动，加强安全生产、食品药品安全监管，社会大局总体稳定。开展新一轮双拥模范城(县)创建活动，军政军民关系进一步密切。创建为国家防震减灾示范城市。工会、妇女儿童、关工委、慈善、老龄事业持续发展，民族宗教、外事侨务、统计、供销、档案、打私、人防、气象等工作取得新成绩。

法治建设有效加强。推进依法行政，设立市政府法律顾问室，清理规范性文件，做好政务公开工作，实现一村(社区)一法律顾问全覆盖，依法行政考评在粤东西北地区排第2名。认真执行市人大及其常委会的各项决议决定，自觉接受人大、政协监督，办理人大代表建议58件和政协提案147件，办复率100%。开展“三严三实”专题教育、“工作落实年”活动，整治公务员队伍不严不实问题，进一步改进政府机关作风，形成良好的干事创业氛围。落实党风廉政建设责任制，加大监察、审计力度，严肃查处违纪违法案件。

各位代表，回顾五年发展历程，我们深刻地体会到：必须把加快发展作为第一要务，增强经济综合实力;必须把改革创新作为不竭动力，增创体制机制新优势;必须把改善民生作为工作出发点和落脚点，才能赢得人民群众的认可、支持和拥护;必须把真抓实干作为推进工作关键，才能确保各项部署落到实处。这些宝贵经验，我们将继续传承和发扬。

各位代表，“十二五”时期成绩来之不易，这是市委科学谋划、正确领导的结果，是市人大和市政协有效监督、大力支持的结果，是历届政府同心同德、艰苦奋斗的结果，是全市人民团结一心、努力拼搏的结果。在此，我代表市人民政府，向全市人民和所有关心支持阳江改革发展的同志们、朋友们，表示衷心的感谢!

回顾五年的工作，我们清醒地看到，全市经济社会发展还存在一些困难和问题，政府工作还存在一些不足，主要表现在：经济总量不大，产业结构不优，现代服务业和先进制造业发展较慢，经济发展质量和效益不高;高新技术企业和创新型人才缺乏，研发投入不足，创新驱动能力弱;城乡之间建设水平存在明显差距，统筹协调推进新型城镇化有待加强;加快发展与资源环境约束的矛盾日益凸显，节能减排和生态保护需要继续加大力度;基本公共服务和社会保障水平不高，保障和改善民生任务依然繁重;政府职能转变有待深化，一些政府工作人员责任意识、法治意识和服务意识还不够强。对这些问题，我们一定高度重视，认真加以解决。

二、“十三五”时期的奋斗目标和主要任务

“十三五”时期，是我市全面建成小康社会的决胜阶段。综观国内外形势，我国仍处于可以大有作为的重要战略机遇期，经济发展长期向好的基本面没有变。我省把创新驱动发展作为核心战略和总抓手，努力补齐粤东西北地区发展的短板，为我市加快发展提供了新的机遇和政策支持。我市发展空间广、发展潜力大，拥有港口优势、资源优势和后发优势，完全有可能在新一轮区域竞争中赢得机会、争先进位。因此，我们要坚定信心，振奋精神，主动适应和引领新常态，积极有为开展工作，努力开创我市振兴发展新局面。

“十三五”时期，我市经济社会发展的指导思想是：高举中国特色社会主义伟大旗帜，以马克思列宁主义、毛泽东思想、邓小平理论、“三个代表”重要思想、科学发展观为指导，深入贯彻习近平总书记系列重要讲话精神，全面贯彻党的十八大和十八届三中、四中、五中全会精神，贯彻落实省委十一届五次、六次全会和市委六届十次全会部署，按照“四个全面”的战略布局，坚持发展第一要务，坚持创新、协调、绿色、开放、共享的发展理念，以提升发展质量和效益为中心，以改革创新为动力，以依法治市为保障，继续实施“三大战略”，突出“三大抓手”，做到“四个更加注重”，大力推进经济结构调整和产业转型升级，构建开放型经济新格局，建设绿色生态美丽家园，切实增进民生福祉，确保全面建成小康社会，开启基本实现社会主义现代化新征程。

“十三五”时期，我市经济社会发展的主要目标是：“一个确保、六个提升”，也就是在确保全面建成小康社会的基础上，实现经济综合实力明显提升，地区生产总值年均增速力争达到10%左右，到2020年达2125亿元，人均生产总值达81000元，城乡居民人均收入比2010年翻一番;实现开放合作水平明显提升，基本形成系统完备、科学规范、运行有效的社会主义市场经济体制和开放型经济格局;实现人民幸福指数明显提升，现行标准下农村贫困人口实现全面脱贫，实现基本公共服务均等化，主要民生指标达到全面小康水平;实现文化发展活力明显提升，社会文明程度和全民文明素质不断提高，建设成为文化事业与文化产业繁荣发展的文化名城;实现生态环境质量明显提升，资源节约型、环境友好型社会建设取得重大进展，单位生产总值能耗水耗以及主要污染物排放控制水平处于全省前列;实现社会治理能力明显提升，社会治理体制逐步完善，初步建成治理体系和治理能力现代化的先进城市。

实现“十三五”时期发展目标，必须全面贯彻落实五大发展理念，坚持创新发展，推进以科技创新为核心的全面创新;坚持协调发展，增强发展的协调性和整体性;坚持绿色发展，巩固和提升生态环境优势;坚持开放发展，提高对外开放的质量和发展的内外联动性;坚持共享发展，着力增进人民福祉。

一是推进经济加快发展，实现经济综合实力明显增强。坚持以经济建设为中心，做优增量、调整存量，促进投资、消费、出口协调拉动，推动经济保持中高速增长。坚持以大项目带动，引导各类资本更多投向新兴产业、“互联网+”、公共服务等领域，形成一批可持续、效益好的新产能。创新消费供给，以扩大服务消费为重点带动消费结构升级，培育消费新热点，促进居民消费。创新外贸商业模式，优化外贸结构，发挥出口对经济增长的促进作用。加大企业扶持力度，促进民营经济发展上新水平。始终抓好招商引资，努力引进一批重大项目，增强发展后劲。

二是推进创新驱动和转型升级，构建三次产业协调发展的现代产业体系。提高自主创新能力，全面推动科技创新、产业创新、企业创新等创新活动，加快实现从要素驱动向创新驱动切换。加快经济结构调整和产业转型升级，积极发展先进制造业，改造提升传统产业，大力发展服务业，推进农业现代化，逐步提高第三产业比重。到2020年，研究与试验发展经费支出占地区生产总值比重达到1.3%;第三产业增加值比重提高到40%。

三是推进城乡统筹发展，形成城乡区域一体化发展的新格局。统筹城市发展空间、规模、产业三大结构和规划、建设、管理三大环节，推动中心城区扩容提质，推进阳江滨海新区建设，加快建成现代滨海新城。促进江城、阳东“一城两区”融合发展。抓好市域副中心建设，推动有条件的中心镇发展成为新型小城市，培育各具特色的工业强镇、旅游名镇、商贸重镇、农业大镇，建成一批美丽乡村，促进城乡一体化协调发展。

四是推进绿色低碳循环发展，建设生态宜居的美丽阳江。严格落实主体功能区规划，严守生态保护红线。推进林业生态建设，加强海洋资源保护，实施山水林田湖生态保护和修复工程，筑牢生态安全屏障。到2020年，森林覆盖率达到61%。促进节能减排和资源节约，推进再生资源体系建设，积极发展循环经济和低碳经济，加快创建国家环保模范城市和低碳生态城市，保护好阳江的青山绿水、蓝天白云，促进人与自然和谐发展。

五是推进改革开放，实现开放发展迈向更高水平。坚持向改革要动力，以经济体制改革为重点，深化重点领域和关键环节改革，使市场在资源配置中起决定性作用和更好发挥政府作用。加快外贸转型升级，培育以技术、品牌、质量和服务为核心的外贸竞争新优势，提升对外贸易国际竞争力。立足粤西，密切与粤西其他市交流合作，对接珠三角，全面融入珠西都市圈，加强与“一带一路”沿线国家、地区经贸往来，努力实现充满活力、互利共赢的开放发展。

六是推进民生持续改善，让人民生活更加幸福。坚持以人为本、公平普惠，更加注重民生改善，实施新时期精准扶贫、精准脱贫攻坚工程，促进就业创业，加快发展教育、医疗卫生、健康养老等民生事业，促进文化繁荣发展，完善社会保障体系，提高基本公共服务均等化水平，让全市人民在共建共享发展中有更多获得感。力争五年新增城镇就业17.5万人，居民人均可支配收入年均增长10%左右。加强和创新社会治理，加快建设法治政府和法治社会，促进社会公平正义，维护社会和谐安定。

三、2016年主要工作安排

2016年是实施“十三五”规划的开局之年，做好今年的政府工作意义重大。综合考虑各方面因素，今年全市经济社会发展的主要预期目标是：地区生产总值增长8.5%左右;规模以上工业总产值增长10%;固定资产投资增长8%;进出口总额增长3%;社会消费品零售总额增长10%;一般公共预算收入增长10%;居民消费价格涨幅预期3%左右;居民收入增长与经济增长基本同步;城镇登记失业率控制在3%以内;节能减排约束性指标完成省下达年度计划。重点抓好八个方面工作：

(一)全力促进经济稳定增长

抓好重点项目建设。全年安排重点建设项目81个，总投资2196亿元，年度投资245亿元;预备重点项目52个，总投资1678亿元。落实领导挂点联系重点项目制度，加强项目跟踪服务，解决项目建设制约难题，提高项目开工率、投产率。推动阳江核电站4号机组等项目加快投产，加快阳江抽水蓄能电站、新兴铸管、维达生活用纸等项目建设，推进粤西农海产品批发市场等项目动工建设，加快华厦阳西电厂5、6号机组等项目前期工作，形成重点项目“开工一批、投产一批、储备一批”的良好格局。

扩大外贸进出口。落实促进外贸稳定增长措施，支持企业参加各类贸易促进活动，巩固欧美等传统市场，拓展东盟等新兴市场。引导外贸企业转型升级，提高五金刀剪、水海产品等出口产品竞争力，扩大高新技术产品出口。加快培育跨境电子商务平台和企业，推动企业开展跨境贸易电子商务出口业务。优化进口结构，促进先进技术设备、关键零部件以及资源性原材料等进口。完善港口公共码头服务功能，推进大通关建设，进一步提高贸易便利化水平。

拓展居民消费。多渠道促进城乡居民增收，提高居民消费能力。引导和促进消费升级，积极培育旅游休闲、文化体育、汽车等消费热点。抓住国家推进宽带提速降费、4G加快推广契机，促进新一轮信息消费。推动企业应用电子商务，发展线上线下联动促销，促进阳货网上行、阳货全国行。加快发展农村电子商务，促进农村商品流通和农村消费。推动房地产业健康发展，合理引导住房消费。

提高招商引资实效。继续加大“1+10”专业招商力度，做好投资环境推介、项目洽谈、跟踪服务等工作，推进产业链招商。抓好先进装备制造业招商，着力引进一批先进装备制造龙头项目和配套项目。落实招商责任，加强招商力量，增强招商引资的针对性、实效性。

促进民营经济发展。加强企业服务，优化民营企业尤其是中小微企业发展环境，推动各类帮扶、激励政策全面落实到民营企业，帮助企业降低成本。支持民营企业参与政府投资项目、重大基础设施项目和公用事业等领域，支持民营骨干企业和后备骨干企业加快发展壮大，激发内源型经济发展潜力。

(二)推进产业转型升级

按照中央和省的部署，积极推进供给侧结构性改革，抓好去产能、去库存、去杠杆、降成本、补短板各项工作。

做大做强临港工业。深入实施工业加快发展和转型升级攻坚战三年行动方案，加快培育发展年产值超500亿元的产业集群。依托三大镍合金企业，引进关联企业项目，推动广青合金热送设备及不锈钢深加工技改等项目建设，打造中国(阳江)高端不锈钢产业基地。着力谋划新的能源项目，推进核电、火电、风电、太阳能光伏等能源项目建设。积极引进原材料及基础件、新能源装备、海洋工程等装备制造企业，提升发展传统机械装备制造业，推动先进装备制造业形成新的增长极。

改造提升传统产业。实施技术改造，支持五金刀剪、食品加工等行业企业技术改造提升，引导80家规模以上工业企业实施技术改造，带动产业转型升级。促进“两化”融合，推广机器人应用，加快智能设备更新，提高传统产业增加值率和劳动生产率。推进互联网+传统产业，培育发展产业新业态，推动传统产业向中高端发展。

大力发展现代服务业。以海陵岛大角湾海上丝路旅游区晋升国家5A级景区为契机，积极引进高端旅游综合体项目，优化景区配套设施和管理服务，加快将海陵岛打造成为国际休闲旅游岛。抓好阳春生态、溶洞观光等旅游开发，加强东平、沙扒等景区设施建设，推动旅游景区(点)提质升级。促进文化与旅游融合，加快文化旅游示范点建设。加强旅游区域合作和宣传促销，发展冬季旅游项目，拓展省外客源市场。结合中心城区扩容提质，布局建设大型城市综合体，打造集旅游娱乐、文化休闲、消费购物于一体的商务集聚区。加快五金刀剪、汽车、建材、农产品等专业市场建设，推进三七物流等项目建设，培育发展现代物流业。支持电子商务平台建设，鼓励发展第三方电子商务平台，加快发展电商产业。依托阳江国际金融中心建设，吸引国内外银行、保险、证券等金融机构进驻。鼓励社会资本进入社会事业领域，加快发展教育、医疗卫生、文化体育、健康养老等服务业。

积极发展现代农业。推进农业产业化，促进农业与二三产业融合发展。推进高标准基本农田和农田水利基础设施建设，优化“菜篮子”基地，促进粮食稳产增产。壮大新型农业经营主体，发展适度规模经营。加快阳东区省级现代农业示范区和阳江(双捷)现代农业产业园等农业园区建设，大力发展特色农业、高效农业、生态农业、观光农业。积极发展深蓝渔业，争取新建50艘钢质渔船，加快深水网箱产业园等高标准养殖示范基地建设，发展水海产品深加工，推动海洋渔业转型发展。动工建设闸坡、溪头现代渔港项目，加快建设现代渔港经济区。加快发展农产品现代流通业和农产品加工业，推进家禽“集中屠宰、冷链配送、生鲜上市”工作。

促进园区扩能增效。拓展产业园区发展空间，推动符合条件的县(市、区)依托省级产业转移园区建设产业集聚地。完善产业转移园区基础配套设施，抓好智慧园区建设，改善园区发展环境。推进园区产业项目建设，促进主导产业发展。培育发展大型骨干企业，力争新增年主营业务收入超10亿元企业3家、超50亿元企业2家。清理闲置土地，盘活存量土地，提高园区集约节约用地水平。

(三)抓好创新驱动发展

培育创新型企业。实施大型骨干企业研发机构全覆盖行动，扶持有条件的企业组建国家级、省级工程技术研究开发中心等研发机构，支持企业建立院士、博士后工作站和科技特派员工作站。依托园区加快孵化器建设，完善孵化链条，培育科技型、创新型企业。加快发展高新技术企业，建立高新技术企业后备库，引导创新资源和创新服务向科技型企业集聚。

加大人才培养和引进力度。深入实施省“扬帆计划”和市六大人才培养工程，加快培养适应我市发展需要的应用型人才和创新型高技能人才。建立人才“柔性引进”机制，完善和落实人才引进优惠政策，积极引进创新创业团队和领军人才，吸引学术带头人、科技型企业家、职业创业者、大学生等到阳江创新创业。促进与发达地区人才交流合作，加强创新人才队伍建设。

营造良好创新环境。设立五大科技专项资金，引导企业加大创新投入。加强协同创新，探索推动企业与高等院校、科研院所共建重点实验室、产学研示范基地，建立技术研究开发战略联盟。发挥五金刀剪产业技术研究院作用，推进五金刀剪、镍合金材料等领域重大科技专项实施，促进行业技术进步。完善科技成果转化机制，促进科技创新与金融、产业融合发展。加强知识产权运用和保护。

(四)加快新型城镇化步伐

推动中心城区扩容提质。落实中央城市工作会议精神，推动以人为核心的新型城镇化。加快滨海新区建设，以金平路(一期)建设为推动，促进新区向南向海拓展。推进高铁站建设，在周边区域统筹布局建设商贸、物流等项目，着力打造高铁商贸集聚区。加快建成阳江国际金融中心，推进“六路一桥”等21个基础设施项目建设，加快新区教育、卫生、市场等14个公共服务配套项目建设，抓好漠阳湖公园(一期)、一河两岸生态景观带、滨海生态公园建设，完善新区发展功能。统筹推进江城、阳东城区建设，健全市政设施和公共服务设施，促进市政管理、公共服务一体化。抓好阳春、阳西、海陵三大市域副中心建设，完善产业、服务、居住和城乡衔接功能。

提升城市规划建设管理水平。抓好城市总体规划和城市基础设施建设“十三五”规划等修编编制工作。以创建国家卫生城市和国家环保模范城市为抓手，推进旧城区改造，建成市区城北污水处理厂，加快阳江森林公园(一期)、南山公园等市政工程建设，完善城市功能配套。探索理顺城市管理体制，提高城市管理执法和服务水平，努力消除各种“城市病”。加大道路交通管治力度，加强公共停车场建设管理，增加和优化公交线路，新增公共汽车100辆，完善城市公共交通设施网络。抓好城中村、城乡结合部等环境综合整治，严厉查处违建行为，改善人居环境。

推进镇街和美丽乡村建设。落实镇(街道)经济社会管理权限，实施差别化的镇域发展政策，全面提升中心镇和重点镇辐射带动能力。推进3个省级新农村示范片和卫生镇村建设，完成100条美丽乡村创建任务，启动新一批美丽乡村建设。

抓好交通等重要基础设施建设。谋划推进新的“一港四路”建设。加快深茂铁路阳江段、汕湛高速公路阳春段等13个续建项目建设，推动云阳高速公路阳春段建成通车。推进中山至阳江高速公路阳春段等9个项目动工。做好粤西沿海高速公路阳江段等7个项目前期工作。推进阳江港码头泊位建设。加快阳江合山机场改扩建。建成 500千伏回隆、220千伏旗鼓岭等输变电工程。加快漠阳江双捷引水工程拦河闸重建、漠阳江中下游综合治理等水利工程建设。推进信息基础设施建设，加快电子政务云平台建设，落实“互联网+”行动计划，推进智慧城市建设。

(五)加大改革开放力度

深化经济领域改革。深入实施企业投资项目负面清单、行政审批清单、政府监管清单。深化预算管理等制度改革。落实国家税制改革措施，推进依法治税。创新投融资机制，在基础设施和公共事业等重点领域推广政府与社会资本合作(PPP)模式。规范各类融资行为，防范非法集资等金融风险。推进国有企业改革，发展混合所有制经济。深化“三证合一”登记制度改革。推进供销社综合改革和国有林场改革。

深化行政管理体制改革。优化政府机构设置、职能配置和工作流程，推进简政放权、放管结合、转变政府职能工作，最大限度减少政府对微观事务的管理。深化行政审批制度改革，落实好市县镇三级政府部门权责清单，继续取消和调整一批行政许可事项。推进镇(街道)行政体制改革，完善基层治理体系。深化事业单位改革，抓好法人治理结构建设。加快行政事业性收费改革。

深化社会体制和农村改革。坚持保基本、兜底线、促公平、可持续，推进社会保障制度改革。深化收入分配制度改革、教育综合改革和医药卫生体制改革。全面实施不动产统一登记制度。抓好户籍制度改革。开展农村社区建设试点工作。推进农村集体产权制度、农业经营制度、农业支持保护制度、城乡发展一体化体制机制和农村社会治理制度5大领域改革。

深化区域合作。积极参与“珠中江+阳江”经济圈建设，主动接轨广东自贸区，积极承接珠三角地区信息、人才、产业等辐射。

主动参与珠江西岸先进装备制造产业带建设，与各市加强产业对接，推动先进装备制造业互补错位发展。加强与珠海市对口共建，继续深化以产业园区、城市建设、民生事业等为重点的合作。推进湛茂阳经济圈建设，加强与湛江、茂名在交通建设、产业发展、港口对接等方面合作。

(六)加强生态环境保护

抓好节能减排。严把环保准入关口，淘汰和控制新建高耗能项目。加强低碳技术应用，抓好工业、交通等领域节能技术改造。提高建筑节能标准，推广绿色建筑和建材。推进循环经济园区建设，加快发展循环经济。实行公共交通优先，推广新能源汽车，鼓励绿色出行。

抓好环境整治。加强漠阳江等重要流域水环境综合整治，清理水库保护区畜禽养殖，保护好水资源。完成11个中心镇生活污水处理厂及配套管网、一般镇生态型污水处理设施建设。加强大气污染防治，推动黄标车、老旧车提前淘汰，抓好高污染燃料锅炉、扬尘等污染防治，严控PM2.5、PM10。整治禁养区、禁建区养殖场，抓好农村生活垃圾收运处理，加强农业面源污染防治。

抓好生态建设。强化环境分区控制，加强生态环境保护。抓好森林碳汇等重点生态工程，推进7个森林公园建设，完成5.05万亩造林任务，建设生态镇、生态村。保护好岸线、海岛、湿地、沙滩等海洋资源，推进美丽海湾建设。严格实施年度土地利用计划，严守耕地保护红线。

(七)切实保障和改善民生

办好十件民生实事。今年计划投入19.55亿元，集中力量办好底线民生、政务便民、精准扶贫、文化惠民等十件民生实事。明确责任分工，强化责任落实，加大工作力度，确保各项任务完成。

提高社会保障水平。加强就业创业政策扶持，鼓励以创业带动就业，促进高校毕业生、异地务工人员、就业困难人员等重点群体就业。强化社会保险扩面征缴。推进机关事业单位养老保险制度改革。推行基本医疗保险异地就医即时结算。继续提高城乡养老保险待遇、低保救助、五保供养等标准。加快建成阳东、阳春、阳西等养老院，扶持和规范民办养老机构建设。扎实开展助残服务工作。抓好保障性住房建设，新建公租房360套，改造农村危房5713户、国有工矿棚户区350户。

全面发展社会事业。巩固省教育强市创建成果，深入实施第二期学前教育三年行动计划，加快义务教育均衡优质标准化发展，巩固提升高中阶段教育普及水平，推进职业教育、继续教育，办好特殊教育，鼓励发展民办教育。继续探索和完善医院托管等模式，深化县级公立医院改革，推动优质医疗资源向基层延伸。稳妥推进市第三人民医院改革搬迁和市中西医结合医院改制，推动社会办医。加快市人民医院新住院大楼、市妇幼保健院新院、市中医医院(二期)和120急救指挥中心建设。抓好重大传染病防控。落实一对夫妇可生育两个孩子政策，改革完善计划生育服务管理，促进人口均衡发展。加快发展文化体育事业，整合资源，升级改造文体设施，加快构建现代公共文化服务体系。实施文化惠民工程，建设20个基层公共文化体育综合服务中心，扎实开展群众文体活动。扶持阳江漆艺等文化产业基地建设，发展阳江特色文化产业。弘扬积极向上的网络文化，净化网络环境。做好“南海Ⅰ号”参与海上丝绸之路申遗工作。动工建设市档案、方志、党史馆。做好市博物馆搬迁工作。推进“七五”普法工作，增强全民守法意识。抓好双拥共建工作，巩固军政军民团结。开展第三次全国农业普查。加强工会维权服务工作。认真解决妇女儿童发展重点难点问题。继续做好民族宗教、外事侨务、人防、气象、打私等工作。

实施精准扶贫攻坚工程。以农村居民人均可支配收入4000元为扶贫标准，将相对贫困户27633户、相对贫困人口62099人作为帮扶对象，重点帮扶88条相对贫困村。坚持规划到户、责任到人，因人因地施策，分类扶持，推动贫困村和贫困户加快实现精准脱贫，力争今年减少相对贫困人口不少于1.3万人。

推进平安阳江建设。抓好“5+5”社会矛盾化解，解决群众合法合理诉求。加强信访信息系统建设，推行网上信访。推进公安机关“四项建设”，创新完善社会治安立体化防控体系建设，强化重点地区和突出犯罪的集中打击整治。实行安全生产党政同责、一岗双责、失职追责，落实企业主体责任，深化重点领域安全生产专项整治。加强食品药品监管，保障公众安全。健全应急管理机制，提高突发事件和自然灾害应急处置能力。

(八)继续加强政府自身建设

依法履行职能。坚持法定职责必须为、法无授权不可为，推进机构、职能、权限、程序、责任法定化。积极配合人大开展地方立法，做好重点领域立法工作。推行政府法律顾问制度，加强行政决策合法性审查，建立重大决策终身责任追究制度及责任倒查机制。坚持严格规范公正文明执法，加强安全生产、环境保护等重点领域执法工作。优化行政服务，完成市、县、镇、村“五统一”政务服务平台建设，着力打造法治化的政务环境、营商环境、人文环境。

强化政务监督。自觉接受人大及其常委会的监督，定期向人大及其常委会报告工作，自觉接受人民政协民主监督，切实做好人大代表议案、建议和政协提案办理工作。广泛听取各民主党派、工商联、无党派人士和各人民团体的意见建议。全面推进政务公开，加强政府网站信息发布和内容建设，加大重点领域政府信息公开力度。健全监察、司法、审计等联动的行政权力监督机制。

持续改进作风。推进作风建设常态化，巩固“三严三实”专题教育成果，整治庸政懒政怠政。发扬严深细实的工作作风，推动政府工作高效落实。加强政府工作督查督办，确保政令畅通。推动建立改革容错免责机制，坚持“三个区分”，鼓励改革、宽容失误，营造干事创业的良好氛围。

加强廉政建设。强化党风廉政建设主体责任，认真执行《中国共产党廉洁自律准则》和《中国共产党纪律处分条例》，推进政府系统党风廉政建设和反腐败工作。完善政府系统行政权力、防治腐败制约机制，强化重点部门和岗位廉政风险防控。实行“一案双查”，严肃查处违法违纪行为，努力建设风清气正、廉洁高效、人民满意的政府。

各位代表!美好蓝图已经绘就，发展前景催人奋进。让我们紧密团结在以习近平同志为总书记的党中央周围，在省委、省政府和市委的坚强领导下，高举中国特色社会主义伟大旗帜，解放思想，开拓创新，团结奋斗，真抓实干，为全面实现今年和“十三五”经济社会发展目标，加快全面建成小康社会而努力奋斗!
